# TR34/L98H Mutation in *CYP51A* Gene in *Aspergillus fumigatus* Clinical Isolates During Posaconazole Prophylaxis: First Case in Korea

**DOI:** 10.1007/s11046-018-0271-8

**Published:** 2018-06-01

**Authors:** Hyeon-Jeong Lee, Sung-Yeon Cho, Dong-Gun Lee, Chulmin Park, Hye-Sun Chun, Yeon-Joon Park

**Affiliations:** 1 Division of Infectious Diseases, Department of Internal Medicine, The Catholic Blood and Marrow Transplantation Center, Vaccine Bio Research Institute, Seoul St. Mary’s Hospital, College of Medicine, The Catholic University of Korea, 222, Banpo-daero, Seocho-gu, Seoul, 06591 Republic of Korea; 2 Vaccine Bio Research Institute, Seoul St. Mary’s Hospital, The Catholic University of Korea, 222, Banpo-daero, Seocho-gu, Seoul, Republic of Korea; 3 The Catholic Blood and Marrow Transplantation Center, Seoul St. Mary’s Hospital, College of Medicine, The Catholic University of Korea, 222, Banpo-daero, Seocho-gu, Seoul, 06591 Republic of Korea; 4 Department of Laboratory Medicine, Seoul St. Mary’s Hospital, College of Medicine, The Catholic University of Korea, 222, Banpo-daero, Seocho-gu, Seoul, 06591 Republic of Korea

**Keywords:** *Aspergillus fumigatus*, Azoles, Posaconazole, *CYP*51A

## Abstract

Azole resistance in *Aspergillus fumigatus* is an emerging problem, especially in immunocompromised patients. It has been reported worldwide, including in Asia, but has not yet been reported in Korea. Here, we report a case of invasive pulmonary aspergillosis (IPA) caused by azole-resistant *A. fumigatus* that developed in a hematopoietic stem cell transplantation recipient during posaconazole prophylaxis for immunosuppressive therapy of graft-versus-host diseases. We identified TR34/L98H/S297T/F495L mutation in the *CYP51A* gene of *A. fumigatus* clinical isolate obtained from bronchial washing fluid. Minimal inhibitory concentrations for itraconazole, voriconazole, and posaconazole were > 16, 1, and 4 μg/mL, respectively. While IPA improved partially under voriconazole treatment, the patient died from carbapenemase-producing *Klebsiella pneumoniae* bacteremia. Further epidemiological surveillance studies are warranted.

## Introduction

*Aspergillus* infections including invasive aspergillosis (IA) mainly affect immunocompromised patients, such as hematopoietic stem cell transplantation (HSCT) recipients or patients undergoing immunosuppressive therapy. Due to the increased number of immunocompromised patients, the incidence of IA has increased over the past three decades. Among *Aspergillus* species (spp.), *Aspergillus fumigatus* remains the most common species in all pulmonary syndromes. Although diagnostic advances and new triazole antifungal drugs have now been established, mortality rates associated with IA remain high and range between 28.5 and 50% [1].

As triazole drugs have been the mainstay of both treatment and prevention of IA since the 1990s, there were concerns about the increasing possibility of that azole-resistant *Aspergillus* spp. or azole breakthrough IA by expanding the usage of triazoles [2]. Since the first azole-resistant *A. fumigatus* was found both in the Netherlands and in Italy in 1998 [3], azole resistance in *A. fumigatus* isolates has been found in almost every European country, the Middle East, Asia, Africa, Australia, and most recently, North and South America. Substitution of leucine 98 with histidine in the *CYP51A* gene in combination with a 34-bp tandem sequence in the promoter gene (TR34/L98H) is a dominant resistance mechanism thought to be acquired from the environment [4–6]. Reported rates of azole-resistant *A. fumigatus* vary from 0.6 to 27.8% [5, 7]. However, there has been no evidence that azole-resistant *A. fumigatus* is a problem in Korea.

## Case Report

A 27-year-old man who received second matched unrelated donor HSCT for relapsed acute lymphoblastic leukemia (ALL) (D + 210) was hospitalized for treatment of aggravated grade IV skin graft-versus-host disease (GVHD). The patient started high-dose steroid therapy (> 1 mg/kg per day of prednisolone) for GVHD and continued taking posaconazole (PCZ) tablets (300 mg q12 h for 2 doses and then 300 mg once daily) which had been administered for 77 days from the outpatient clinic for fungal prophylaxis in severe GVHD. During the high-dose steroid treatment, more than 10% of blasts were detected in peripheral blood cell counts, and ALL again relapsed after the second HSCT was confirmed.

**Fig. 1:**
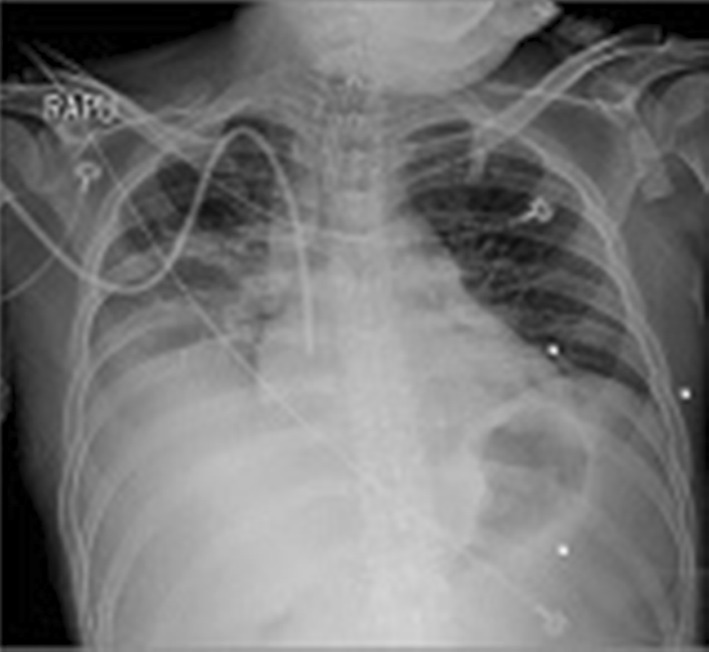
Chest X-ray showed infiltrations in right upper and lower lung fields

**Fig. 2:**
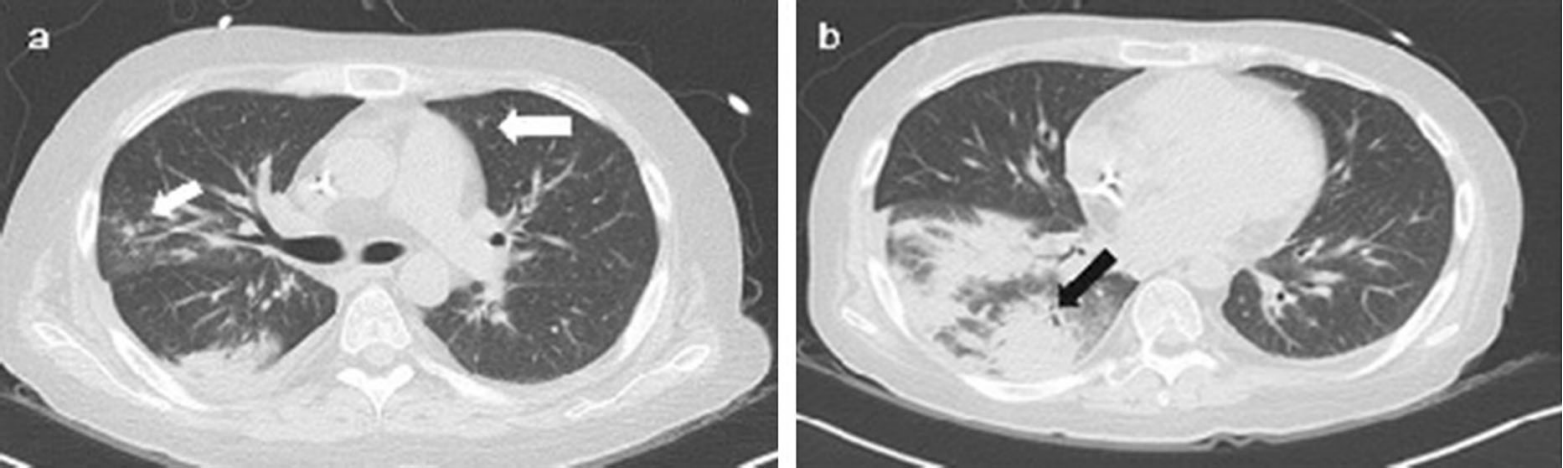
Low-dose chest computed tomography. **a** Ill-defined centrilobular nodules in both lungs (white arrows). **b** Peribronchial consolidation that accompanied by ground-glass opacities

On the 7th day of hospitalization (HD 7), the patient suddenly complained of fever (maximum body temperature 38.6 °C) and dyspnea, and then, his blood pressure dropped to 84/43 mmHg. Oxygen demand was gradually increased, and tracheal intubation was performed. At that time, it was the seasonal influenza epidemic, and rapid influenza antigen test resulted in influenza A positive and chest X-ray showed infiltrations in the right lung fields (Fig. 1). Therefore, the first impression was influenza A pneumonia with septic shock. Considering the possibility of combined other nosocomial bacterial pneumonia or atypical pneumonia, not only peramivir (600 mg once), but also cefepime (2g q12 h), levofloxacin (750 mg once daily), and teicoplanin (400 mg q12 h for 3 doses and then 400 mg once daily) were administered. On the chest, low-dose computed tomography (LDCT) performed, and multifocal ground-glass opacities (GGOs) accompanied by peribronchial consolidations and ill-defined centrilobular nodules in both lungs were observed (Fig. 2). On the 3rd day of fever onset (HD 10), bronchoscopy was performed. His condition recovered rapidly and intubation was removed on the 4th day. Blood and sputum cultures, *Streptococcal pneumoniae* and *Legionella* urinary antigen tests, *Mycoplasma* serum IgM/IgG tests, and serum galactomannan assay were all negative.

On the 4th day after bronchoscopy (HD 11), *Aspergillus* spp. was cultured from the bronchial washing fluid specimen. It was thought to be a true pathogen because chest LDCT revealed consolidations with surrounding GGO and nodules consistent with fungal pneumonia that developed during the course of long-term maintenance of high-dose steroid treatment. According to revised European Organization for Research and Treatment of Cancer/Mycosis Study Group (EORTC/MSG) criteria [8], the patient was diagnosed as a culture-positive invasive pulmonary aspergillosis (IPA) with probable category. At the time of diagnosis of IPA, the patient had been receiving PCZ for 87 days, maintaining a therapeutic range of PCZ serum concentrations (1048–2232 ng/mL). Therefore, this case was considered as PCZ breakthrough IA.

PCZ was changed to intravenous voriconazole (VCZ) (loading dose 6 mg/kg q12 h, then 4 mg/kg q12 h). Other antibiotics were also discontinued because there was no evidence of other bacterial pathogens. Eight days later, *Aspergillus* spp. was finally confirmed as *A. fumigatus* by internal transcribed spacer (ITS) sequencing and PCR of the β-tubulin gene as follows. Their entire ITS regions were amplified using the primers of ITS1-F_KYO2 (5′-TAGAGGAAGTAAAAGTCGTAA-3′) and ITS4 (5′-TCCTCCGCTTATTGATATGC-3′), as previously described [9]. β-tubulin PCR was performed by bt2a (5′-GGTAACCAAATCGGTGCTGCTTTC-3′) and bt2b (5′-ACCCTCAGTGTAGTGACCCTTGGC-3′) [10]. Amplicons of ITS and β-tubulin were sequenced and then identified using the BLASTN. Antifungal susceptibilities were determined using the broth dilution method, as recommended by the Clinical and Laboratory Standards Institute (CLSI) M38-A2 (2008) [11]. Antifungal susceptibility testing of the *A. fumigatus* revealed high minimal inhibitory concentrations (MICs) to both itraconazole (ITZ) and PCZ (MICs were > 16 and 4 μg/mL), while VCZ MIC revealed a susceptible upper limit (1 μg/mL). Chest X-ray showed steady improvement, and intravenous VCZ was changed to VCZ tablets (200 mg q12 h) and maintained. Therapeutic drug monitoring of VCZ was performed every week, and it was maintained within therapeutic range between 1.2 and 4.2 μg/mL. Thereafter, the patient’s absolute neutrophil counts were declined, and he experienced repeated neutropenic fever. On the 69th day of hospitalization (HD 69), the patient died due to *KPC*-producing carbapenem-resistant *Klebsiella pneumoniae* bacteremia which was thought to be associated with concurrent gut GVHD. Previous lesions of IPA were decreased in size (from 5.4 to 1.3 cm) at that time. Subsequently, the *A. fumigatus* isolate was analyzed for any known azole-resistant mutations in *CYP51A* gene. The amplification and sequencing of *CYP51A* promoter were performed using AFTR-F (5′-TAATCGCAGCACCACTTCAG-3′) and AFTR-R (5′-GCCTAGGACAAGGACGAATG-3′) [12]. Their *CYP51A* and promoter sequences were compared to that of an azole-susceptible *A. fumigatus* strain (GenBank accession no. AF338659). Including TR34/L98H, multiple mutations including S297T in the *CYP51A* gene were identified as shown in Table 1.

**Table 1:** In vitro antifungal susceptibility profile and *CYP51A* amino acid substitutions in *A. fumigatus* isolates in this case

Source of isolate	PCZ exposure (days)	Antifungal MIC (μg/mL)				MEC	*CYP*51A amino acid substitutions		
		ITZ	VCZ	PCZ	AMB	CAS	TR34	Codon98	Others
Bronchial washing fluid	87	> 16	1	4	0.5	0.06	+	L98H	Y46F, V172 M, T248 N, E255D, S297T, K427E, F495L

*PCZ* posaconazole, *MIC* minimum inhibitory concentration, *MEC* minimum effective concentration, *ITZ* itraconazole, *VCZ* voriconazole, *AMB* amphotericin B, *CAS* caspofungin

## Discussion

This case is that of culture-positive IPA with probable category in an HSCT recipient, which developed during PCZ prophylaxis due to GVHD. Isolated *A. fumigatus* showed a high MIC for ITZ and PCZ, and *CYP51A* gene analysis identified a TR34/L98H mutation. In Korea, antifungal susceptibility epidemiology studies in *Aspergillus* spp. [13] have been reported, but no known gene mutation has been found. This is the first case of IPA caused by TR34/L98H azole-resistant *A. fumigatus* in Korea.

Based on a systematic study of azole resistance rates and mechanisms conducted by the Nijmegen group in the Netherlands, TR34/L98H is the most common mutation found in clinical and environmental azole-resistant *A. fumigatus* isolates across Europe [7, 14]. Environmental isolates (soil samples from gardens, plant seeds, hospital surroundings, aerial samples of hospitals) harboring TR34/L98H exhibit resistance to azole fungicides, which are chemically similar to medical triazoles, showing cross-resistance [15]. Therefore, azole fungicides used in the environment have been suggested to induce azole resistance of *Aspergillus* spp. clinical isolates [4, 15]. Furthermore, close genotypic accordance between *A. fumigatus* environmental and clinical isolates has been demonstrated, and many patients harbor a single dominant resistance mechanism, even though they are azole-naive or epidemiologically unrelated [4, 6, 15]. Therefore, it has been suggested that the main route of resistance is an acquisition from environmental source [4]. Strains with the TR34/L98H mutation of *CYP*51A gene also frequently harbor non-synonymous mutations in the *CYP51A* gene. Some of them are presented alone or in combination, which are known to be associated with higher MICs than the wild type [5, 7].

In this case, isolated *A. fumigatus* showed relatively higher PCZ MIC (4 μg/mL) and lower VCZ MIC (1 μg/mL) compared to previously reported MICs of TR34/L98H strains [16]. Also, multiple amino acid substitutions including S297T (Table 1) were found in our isolate. Although most of these substitutions are not clearly characterized with phenotypic relationships, a recent report has suggested that S297T substitution in TR34/L98H strains might represent a compensatory mutation showing this low VCZ MIC [17]. Although there have been no data on the azole resistance of *A. fumigatus* environmental and clinical isolates in Korea, our hypothesis on the resistance mechanism of the ITZ- and PCZ-resistant strain in this case is a combination of environmental acquisition and the acquired resistance from the patient, probably due to long-term PCZ exposure.

In a national survey conducted on aspergillosis in the Netherlands, where epidemiology data are relatively well established, azole resistance rates have been reported between 5 and 10%, and up to 30% in high-risk wards [18]. VCZ is still the first drug of choice of IA, even in the clinical setting of high resistance. There are no clear guidelines for azole-resistant and/or breakthrough IA [18]. There are only expert opinions that change the antifungal class to another or the combination of VCZ plus echinocandin when the resistance threshold exceeds 10% or in the case of azole breakthrough IA [1, 18, 19].

Interestingly, this is an IPA case accompanying influenza A pneumonia, either. Influenza has been established a risk factor for IA since 2009 pandemic influenza A/H1N1 based on many case reports [20–24]. Influenza-associated IA has also been reported in Korea, even in immunocompetent patients [22, 23]. The interval between the diagnosis of influenza and that of IA varies, and some cases have been diagnosed almost simultaneously as in this case. Although a clear mechanism between influenza and IA has not yet been elucidated, cell-mediated defects, disruption of normal ciliary clearance, or virus-induced host adaptive immunity deficiency model has been proposed [22–25]. Influenza-associated IA showed high mortality rate of 47–61% in a recent study [24]. However, the impact of concomitant influenza pneumonia with IPA on mortality is not clear in this case, since the direct cause of death appears to be the *K. pneumoniae* bacteremia lasted until the day before death.

Mortality rates of patients with culture-positive azole-resistant IA have been reported as 50–100%, higher than those of azole-susceptible IA [4, 5, 26]. In addition, there are only limited treatment options in cases where azole breakthrough IA is increasing as in the present case. Currently, *A. fumigatus* susceptibility tests including MICs and *CYP*51A mutations are not routinely available in most clinical settings. Therefore, azole resistance prevalence surveillance studies are needed for establishing the best choice of treatment. In Korea, the first case has been reported, and further epidemiological surveillance studies are warranted.
